# Robust Template Matching Using Multiple-Layered Absent Color Indexing

**DOI:** 10.3390/s22176661

**Published:** 2022-09-03

**Authors:** Guodong Wei, Ying Tian, Shun’ichi Kaneko, Zhengang Jiang

**Affiliations:** 1 School of Computer Science and Technology, Changchun University of Science and Technology, Changchun 130022, China; 2 Graduate School of Information Science and Technology, Hokkaido University, Sapporo 060-0814, Japan

**Keywords:** color features, absent colors, apparent colors, total color space, multiple-layered structure, margin

## Abstract

Color is an essential feature in histogram-based matching. This can be extracted as statistical data during the comparison process. Although the applicability of color features in histogram-based techniques has been proven, position information is lacking during the matching process. We present a conceptually simple and effective method called multiple-layered absent color indexing (ABC-ML) for template matching. Apparent and absent color histograms are obtained from the original color histogram, where the absent colors belong to low-frequency or vacant bins. To determine the color range of compared images, we propose a total color space (TCS) that can determine the operating range of the histogram bins. Furthermore, we invert the absent colors to obtain the properties of these colors using threshold $h_{T}$. Then, we compute the similarity using the intersection. A multiple-layered structure is proposed against the shift issue in histogram-based approaches. Each layer is constructed using the isotonic principle. Thus, absent color indexing and multiple-layered structure are combined to solve the precision problem. Our experiments on real-world images and open data demonstrated that they have produced state-of-the-art results. Moreover, they retained the histogram merits of robustness in cases of deformation and scaling.

## 1. Introduction

Robust matching and searching are challenging problems in image processing [1,2]. These challenges include rotation [3], deformation [4], scale variations [5], partial occlusion [6], and illumination variation [7]. Existing matching and searching algorithms attempt to solve these challenges by a single feature or complex feature combinations [8,9]. However, accurately and robustly matching the background clutter and discriminating similar objects are difficult. In this study, we aim to estimate the location of a target in an image sequence with strong robustness and high precision, particularly in the case of rotation, deformation, and scale variations.

A color histogram is a statistical measure of color feature distribution in an image. The merits of using color histograms are non-complicated computing processes and robust to deformation and scale variation in template matching [10,11,12]. Owing to the lack of location information, most color histogram-based algorithms can determine the target in the searched image; however, they still have a precision problem when compared with the correct location. Utilizing the characteristics of template matching is a breakthrough. The earlier popular and effective color histogram-based algorithms for template matching, such as color indexing (CI) and cumulative color histogram (CCH) [13,14], search a target location in given color space by different similarity measures. They can work well with the change in deformation and scale variation. However, the ability to handle noise and illumination is difficult. Han et al. [15] proposed the fuzzy color histogram to solve sensitive problems. It involved an adjustable fuzzy membership matrix to process noise interferences and illumination variation. Therefore, the distinguishable is reduced between similar objects. The above-mentioned color histogram-based methods have two common points. The first one is that just use color histograms to match or search. The other one is that it focuses on bins with high contribution or frequency to design similarity measures.

The conventional template matching algorithms include Sum-of-Squared-Difference (SSD), Mean-Absolute-Differences (MAD), and Normalized-Cross-Correlation (NCC). They have a long history of image processing, whereas they can not deal with complicated conditions, especially deformation and scale variations. Because SSD, MAD, and NCC are corresponding all pixels based on the location relationship between the template image and compared image in the calculation process. Recently, the Best-Buddies-Similarity (BBS) [16,17] is introduced for template matching to solve this issue. It divided the template image into blocks, and then the number of Best-Buddies Pairs is counted between the template and target images. Each pair of blocks should correspond based on the theory of the Nearest-Neighbor. Therefore, BBS is a two-directional comparison for the Nearest-Neighbor. BBS overcomes the demerit of typical template matching approaches which counted all pixels within the template or target image to measure their similarity. This is unreasonable in many cases. For example, the same target is presented in a different background, or deformation has appeared. In these cases, conventional template matching approaches may lead to false detection. BBS relies on block-to-block matching to find the best-buddies pair to avoid the limitation of using all pixels for calculating. However, when the target has rotational deformation or changes in scale, it is difficult to find an effective best-buddies pair for BBS. Therefore, the challenges of deformation and scaling still have shortcomings for BBS. Next, the Deformation Diversity Similarity (DDIS) [18] is proposed to improve the deficiencies of BBS. Two essential properties are introduced in DDIS. One is the form of Nearest-Neighbor. The second one only needs a single direction to find the best-matched Nearest-Neighbor block to solve the deformation issues. It effectively maintains the advantages of BBS. Meanwhile, finding the best-matched Nearest-Neighbor block in a single direction can reduce the computational complexity. Thus, DDIS is more robust than previous algorithms. However, DDIS is also hard to deal with the problem of scale variation.

Cheng et al. [19] introduced the Quality-Aware template matching (QATM), which focuses on the soft-ranking of the quality of a matching pair. The different cases, such as 1-to-1, 1-to-many, many-to-many, and no-matching, can obtain different values. QATM may easy to deal with different experimental scenarios. However, it is still a challenge for matching the target that is scaling or deformation. Two-stage object detection approaches (Siam R-CNN) are proposed for tracking [20]. This approach is combined with a tracklet-based dynamic programming approach. The merit is it adopts the information of the first frame and previous frame to handle the target occlusion challenge faced by long-term tracking. The demerit is a lot of training is required for different datasets, which increases the computation cost.

In contrast to our prior work [21,22,23], instead of directly exploiting all pixels as color features, we select two other inner layers as candidates to enhance color features. Here, we analyze the color distribution in the given dataset to determine the maximum range relationship of each color channel called total color space (TCS). Next, the multiple-layered matching (ML) is introduced as an improvement based on our earlier absent color indexing (ABC) concept, which compared each layer from the reference image and target image in TCS.

In this paper, we propose the ABC-ML to combine the benefit of the color histogram with template property. A key feature of ABC-ML is that the isotonic principle, that is, the center location of the bounding box of the same target is not to change with partial occlusion, rotation, scale variations, and deformation. The other point is to keep integer multiple relationships based on the number of pixels in each layer. Hence, ABC-ML can retain the merits of ABC. In addition, it is different from the approaches of the pixel-wise and blocks to determine the position information, ABC-ML splits the template image into three layers to keep the central location the same to overcome the existing issue of the drift phenomenon in color histogram-based matching approaches.

The main contributions of this paper are summarized: First, we introduce a concept of TCS on color histogram which defines an accurate range of using color histogram bins. Second, a novel multiple-layered matching is proposed against the drift problem in color histogram-based approaches, which is a pure way to use color histograms and combined them with different layers to add location information and achieve experiment results. This paper is organized as follows: Section 2 introduces new content about the TCS. Section 3 describes absent color indexing and a technique to get threshold by a mean color histogram. Section 4 illustrates the multiple-layered matching approach and how to determine the position and size in each layer. Section 5 includes the experiments to demonstrate the ability of ABC-ML and analyzes the results of experiments. Conclusions and future works are described in Section 6.

## 2. Total Color Space

Color space is widely used in the field of image processing [24,25,26]. Here, we select CIE L*a *b* color space as a candidate which is used in the proposed approach. CIE L*a *b* has a concentrated trend in the aspect of color distribution. It is also close to human vision. Therefore, the application of CIE L*a *b* in ABC-ML is effective. In general, a one- or multi-dimensional color histogram is generated via selected color space. In color space, a color range is an area that expresses the composition of colors in a given color space. A larger color range represents more colors. However, we can find that the maximum and minimum color ranges of each channel obtained in different experimental datasets are inconsistent. Furthermore, it is not necessary to use the entire range in a given color space for statistics on the histogram. Therefore, TCS is defined that is minimum usable or effective range. It enables a better classification of colors without increasing the amount of computation cost.

**Figure 1:**
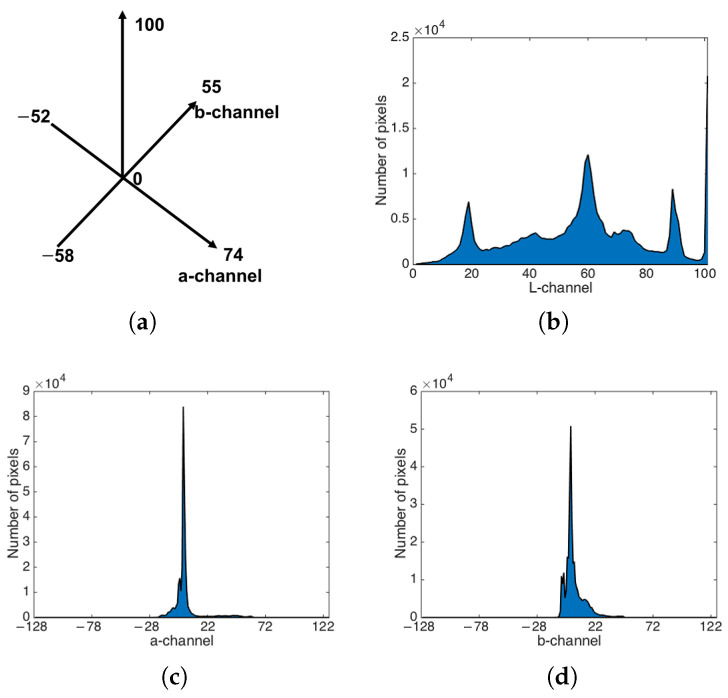
Example of TCS in Girl2 dataset. (**a**) Total color space. (**b**) Color distribution in L-channel. (**c**) Color distribution in a-channel. (**d**) Color distribution in b-channel.

Figure 1 shows the range of colors in different channels and pixel distributions by training the TCS of Girl2 data [27] in CIE L*a *b* color space, where Girl2 data are from OTB-2015. In CIE L*a *b* color space, the range of each channel is (0, 100), (−128, 127), and (−128, 127) in L*, a*, and b* channel, respectively. We analyze all of the images from Girl2 dataset to get the range of each channel. After observing, only the L* channel can keep the same range, and the range of a* and b* channels are (−52, 74) and (−58, 55). It is more precise than initial range. Training of TCS is valuable to reduce the time consumption and to get the minimum usable range of different channels. We also can utilize the TCS in real-time projects by updating them frame by frame.

## 3. Absent Color Indexing

In this section, we first present our algorithm to explain the apparent colors and absent colors. Next, we introduce how to get threshold by the mean color histogram. In final, we use similarity measurement to calculate matched results. To solve the illumination problem, we obtain a two-dimensional color space by transforming RGB images to CIE L*a *b* color space and removing the L* channel, where a* and b* channels are utilized to establish a two-dimensional color histogram in TCS.

### 3.1. Methodology

**Figure 2:**
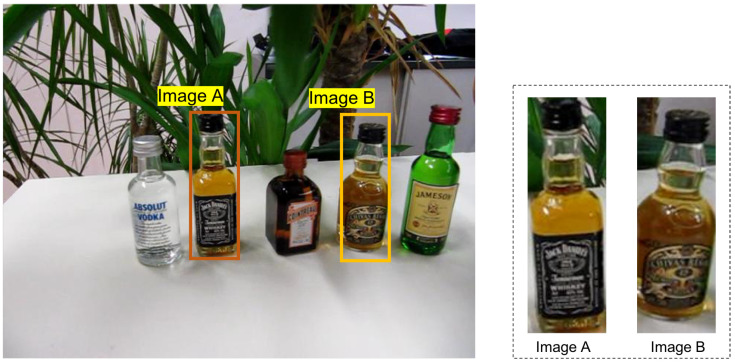
Images A and B are templates with the size 195 × 65.

Figure 2 shows two template images A and B.

We set $\beta_{1}$ and $\beta_{2}$ bins from a* and b* channels by TCS. Two-dimensional color histograms are defined as $P={\left\{p_{ij}\right\}}_{\left(i,j\right)=\left(1,1\right),\cdots ,\left(\beta_{1},\beta_{2}\right)}$ and $Q={\left\{q_{ij}\right\}}_{\left(i,j\right)=\left(1,1\right),\cdots ,\left(\beta_{1},\beta_{2}\right)}$ to represent the histogram form of images A and B. Among them, the total number of pixels in each image is uniform. Moving forward, *P* is used as an example to illustrate the specific algorithm flow.

**Figure 3:**
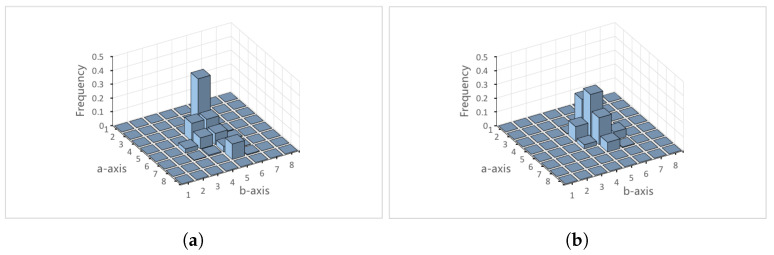
Original color histograms ($\beta_{1}$ = 8, $\beta_{2}$ = 8). (**a**) Histogram *P* for image A. (**b**) Histogram *Q* for image B.

Figure 3 shows the generated histograms *P* and *Q* for images A and B, respectively. We separete original color histogram *P* into two-dimensional color histograms $P^{D}=\left\{p_{ij}^{D}\right\}$ and $P^{\prime }=\left\{p_{ij}^{\prime }\right\}$, where $P=P^{D}+P^{\prime }$. To ease of explanation, the subscripts *i* and *j* are omitted to avoid confusion.

(1) $$p^{D}=p\times \left(1-\phi \left(p\right)\right)=p\times \bar{\phi (p)},$$

(2) $$p^{\prime }=p\times \phi \left(p\right),$$

where ϕ(·) is an indicator function. If $p\leq h_{T}$, ϕ(p)=1; when $p\geq h_{T}$, ϕ(x)=0. $h_{T}$ as a special parameter is introduced in Section 3.2. $P^{D}$ belongs to apparent color histograms including the frequency of major colors in image A. $P^{\prime }$ is the absent color histograms that contain low and zero frequencies. We defined minor colors as absent colors because the property of colors itself is infrequent in color images. The structure of $P^{D}$ and $P^{\prime }$ are same to two-dimensional histogram *P*, where the elements $p^{D}$ and $p^{\prime }$ represent the color frequencies. It is desirable to systematically utilize the information contained in the low frequencies in the histogram through the decomposing process.

**Figure 4:**
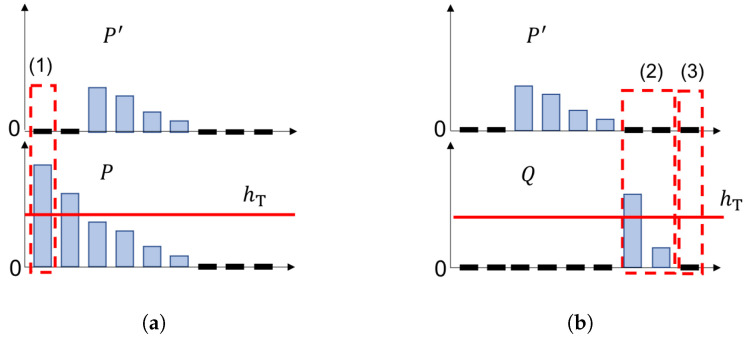
Explanation of how to deal with zero frequency $p^{\prime }=0$ in the inverting process. (**a**) shows the case (1) when $p^{\prime }=0$ and $p>h_{T}$. (**b**) shows the cases (2) and (3) when $p^{\prime }=0$, p=0 and q>0 or q=0.

Next, an reverse operation is executed from absent color histogram $P^{\prime }$ that is a complementary feature for given histogram *P*. However, some special cases should be considered that how to treat zero frequency cases. Assume that $P^{\prime }$, *P*, and *Q* are one-dimensional histograms as shown in Figure 4 to easily explain zero frequency cases, such as $p^{\prime }=0$, during the inversion process of the absent color histogram. $P^{{}^{\prime }}$ is the absent color histogram from histogram *P*. *P* and *Q* are compared histograms, where *Q* is a vital reference for judging whether the zero bins from absent color histogram $P^{\prime }$ need to be reversed. In Figure 4, the first case (1) in Figure 4a describe if $p^{\prime }=0$ and $p>h_{T}$, then $p^{N}=0$; the second case (2) in Figure 4b, if $p^{\prime }=0$, p=0 and q>0, then $p^{N}=h_{T}$; and the last case (3) in Figure 4b, if $p^{\prime }=0$, p=0 and q=0, then $p^{N}=0$. We summarize these three cases in Table 1 to make a clear explanation.

**Figure 5:**
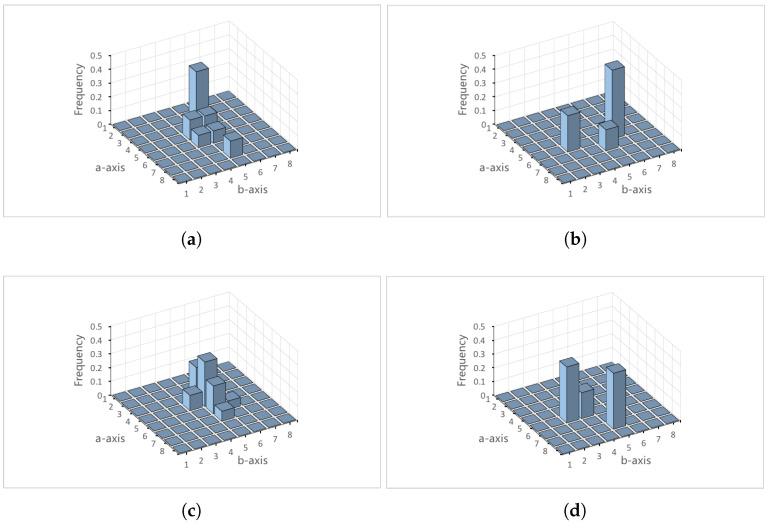
Apparent and absent color histograms. (**a**) $P^{D}$ for image A. (**b**) $P^{N}$ for image A (**c**) $Q^{D}$ for image B. (**d**) $Q^{N}$ for image B.

In Equation (3), we define $P^{N}=\left\{p_{ij}^{N}\right\}$ is the absent color histogram after inverting by abovemention to represent minor or zero frequencies.

(3) $$p^{N}=\left(h_{T}-p^{{}^{\prime }}\right)\phi \left(p\right)\psi \left(p\right)+h_{T}\bar{\psi (p)}\psi \left(q\right).$$

where ψ(·) is another indicator function that satisfies the following conditions, such as p>0, ψ(p)=1; otherwise, if p≤0, ψ(p)=0. Finally, it is necessary to normalize both $P^{D}$ and $P^{N}$ to satisfy the condition that all components should sum to 1. Apparent color histograms $P^{D}$ and $Q^{D}$ and absent color histograms $P^{N}$ and $Q^{N}$ from image A and B are shown in Figure 5, respectively. Note that the initial values of the other elements without any indication were set to 0 in the above equation description.

### 3.2. Threshold Selection

The threshold $h_{T}$ has one of the main roles for definition the apparent and absent colors. We discuss in this section how to define it for making a meaningful algorithm and its effectiveness performance. Mean color histogram, *M* in short, is introduced to obtain an average tendency of color distribution by two color histograms. Meanwhile, it is also utilized to realize a stable definition of threshold $h_{T}$. $M={\left\{m_{ij}\right\}}_{\left(i,j\right)=\left(1,1\right),\cdots ,\left(\beta_{1},\beta_{2}\right)}$ is defined as

(4) $$m_{ij}=\frac{p_{ij}+q_{ij}}{2}.$$

Mean color histogram as a medium is a vital stage before threshold selection. The proportion of each color in the color histogram is analyzed from a statistical perspective in the matched two images. Consequently, we improved the rationality and dynamics of the threshold selection. As a result, the accuracy of the final similarity measurement is ensured. In Equation (5), two-dimensional histogram *M* is converted to another descending order sorted one-dimensional histogram $M^{\mathrm{sorted}}$ as follows:

(5) $$M^{\mathrm{sorted}}=\left\{m_{i-1}^{\mathrm{sorted}}\geq m_{i}^{\mathrm{sorted}}\right\}.$$

**Figure 6:**
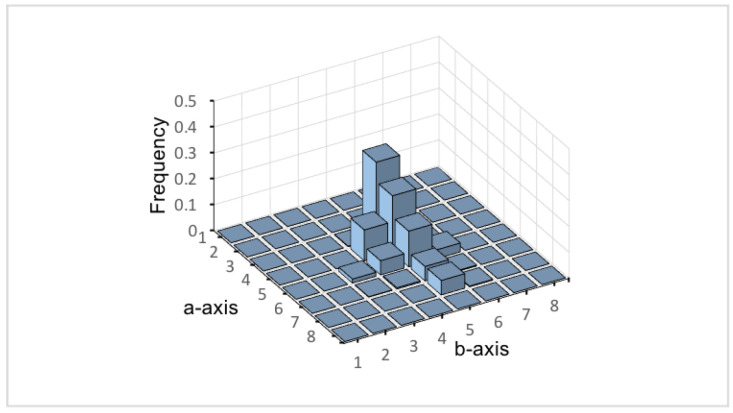
Mean color histogram.

The threshold value $h_{T}$ is described by the following equation. It can separate the set of all of the bins into two sets, apparent and absent colors, in consideration of how rare the absent colors are in the images.

(6) $$\begin{array}{c} h_{T}=\frac{m_{s}^{\mathrm{sorted}}+m_{s+1}^{\mathrm{sorted}}}{2}. \end{array}$$

where $m_{s}^{\mathrm{sorted}}$ and $m_{s+1}^{\mathrm{sorted}}$ are the frequencies of adjacent bins in one-dimensional histogram $M^{\mathrm{sorted}}$. *s* and s+1 represent the order index of bins. *s* is defined as

(7) $$\begin{array}{c} s=\min\left\{n|\sum_{i=1}^{n}m_{i}^{\mathrm{sorted}}\geq 1-\alpha \right\}. \end{array}$$

where α is a significant rate of absent colors, which is used to control the proportion of absent colors in the histogram. The larger α can obtain more absent colors. 1−α is the significant rate of apparent colors. $\sum_{i=1}^{n}m_{i}^{\mathrm{sorted}}$ represents the cumulative frequency of bins from 1 to *n*. *s* is the minimum value of *n* that satisfies the cumulative frequency greater than 1−α. Hence, if we give a value of α, *s* can be found through Equation (7), and then $h_{T}$ is calculated through Equation (6). By using α to dynamically calculate the threshold $h_{T}$, we can perform a stable and efficient decomposition in the range of TCS in comparison with a constant threshold. In the definition of the absent color histogram, since zero frequency has a part of important role for neglecting any effect of noises, we need to remove those frequencies close to zero in the absent color histogram. In our experiments, we define the bins that frequency is less than $0.2\times h_{T}$ in the color histogram as noise. Figure 6 shows the mean color histogram by using histograms *P* and *Q*.

**Figure 7:**
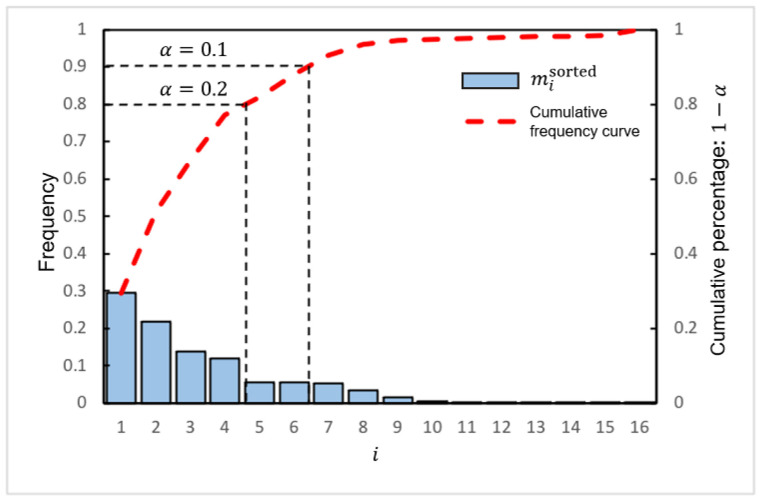
Pareto chart of parameter α and sorted histogram $M^{\mathrm{sorted}}$.

Figure 7 is a Pareto chart [28].

It shows that the value of s is determined by the significant rate α to represent the effectiveness and rareness of absent colors. Next, *s* is utilized to design the threshold $h_{T}$.

### 3.3. Intersection

Histogram matching algorithms depend mainly on similarity measures to match a histogram to other histogram. One of simple similarity measurements is intersection [29], and it has been used in many algorithms and applications. Here, we utilized two color histograms *P* and *Q* in TCS as

(8) $$\begin{array}{c} I\left(P,Q\right)=\sum_{\left(i,j\right)=\left(1,1\right)}^{\left(\beta_{1},\beta_{2}\right)}\min\left\{p_{ij},q_{ij}\right\} \end{array}$$

According to two types of histograms proposed in this paper, we scheme to combine the two intersections from different types of histograms into one through weighting coefficients.

(9) $$S=w_{D}I\left(P^{D},Q^{D}\right)+w_{N}I\left(P^{N},Q^{N}\right)$$

where $w_{D}$ and $w_{N}$ are weights by the constraint $w_{N}=1-w_{D}$. Absent color indexing provides a fair way to treat apparent colors and absent colors, and weighting coefficients are a bridge to connect each type of color histogram.

## 4. Multiple-Layered Matching

Histogram-based matching algorithms have some good properties against rotation, deformation, and scaling. Because these approaches focused on the statistical distribution of given data. Therefore, the reduction of matching precision becomes a common issue.

**Figure 8:**
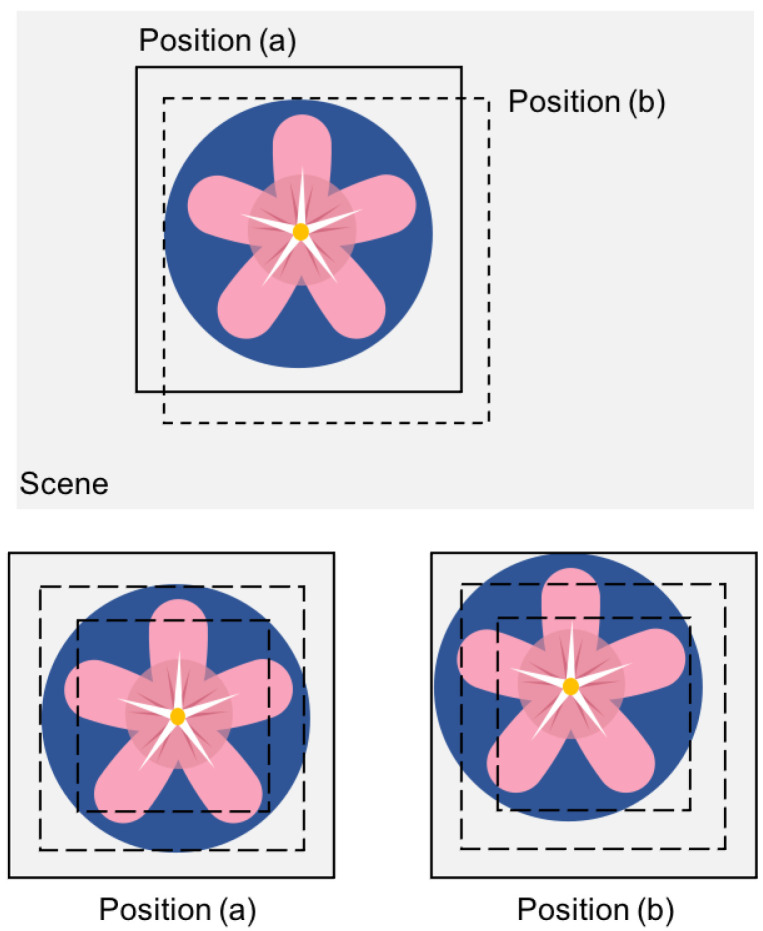
Analyze the color feature distribution at different positions under the multiple-layered structure.

**Figure 9:**
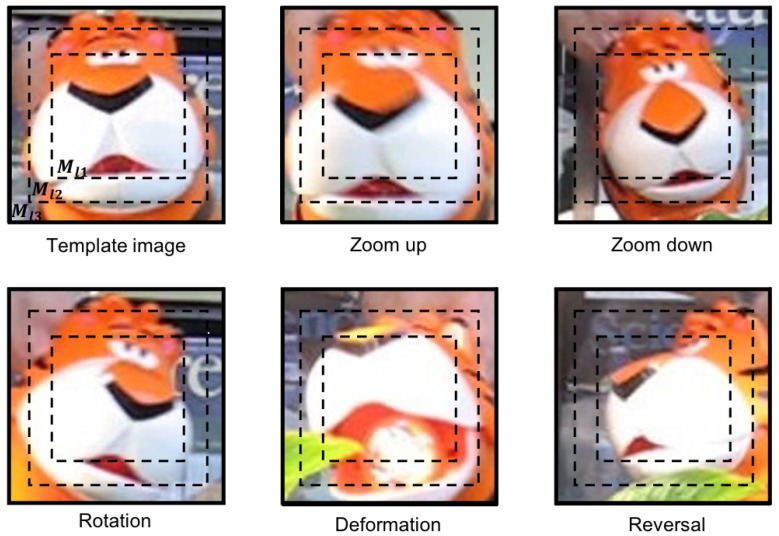
The concept and merit of multiple-layered structure.

For template matching algorithms, they utilize the form of pixel or pixel blocks to compare that can well-controlled precision issues. However, if the case of deformation or scaling is produced during the movement, template matching approaches are difficult to ensure the matching precision. We introduce the demerit of color histogram-based approaches and the merit of combining with multiple-layered matching as shown in Figure 8. In the previous color histogram-based approaches, we may obtain the same similarity when we compare two images in positions (a) and (b). Observing the positions (a) and (b), the feature of color distributions are exactly the same. That is why color histogram-based approaches have weaknesses in matching precision. To solve the offset problem in the matching process, we improve our proposed absent color indexing to combine with the merit of template matching approach through layering the image. Thereafter, multiple-layered struture is proposed as shown in Figure 9, which is based on the isotonic principle to keep the location of the center not changed.

Figure 9 shows when the object occurs rotation, scaling, and deformation during the movement, image features in the central region are kept especially color features. Hence, a multiple-layered structure that divides an image into three layers can be used to obtain the position information, where each layer should restrain the other two layers’ positional relationship. Meanwhile, we can get a more optimized effect in the matching result. In Figure 9, the inner layer is $M_{l1}$ including $p_{m1}$ pixels in the rectangle; the second and third layers are $M_{l2}$ and $M_{l3}$ where they have the relationship as $p_{m2}=2\times p_{m1}$ and $p_{m3}=3\times p_{m1}$.

Assume that, the whole template image is [0,0] and [h,w] in position and size. The upper left of the image is the initial position [0,0]. Next, we should follow below two conditions to satisfy the multiple-layered structure. One is the center point is consistent in each layer; the other is each layer in pixels has the relationships: $p_{m2}=2\times p_{m1}$ and $p_{m3}=3\times p_{m1}$. As an example, the position and size of template image in $M_{l2}$ layer are $\left[\left(1-\frac{\sqrt{3}}{3}\right)\times \frac{h}{2},\left(1-\frac{\sqrt{3}}{3}\right)\times \frac{w}{2}\right]$ and $\left[\frac{\sqrt{3}}{3}h,\frac{\sqrt{3}}{3}w\right]$, and the $M_{l3}$ layer is $\left[\left(1-\frac{\sqrt{6}}{3}\right)\times \frac{h}{2},\left(1-\frac{\sqrt{6}}{3}\right)\times \frac{w}{2}\right]$ and $\left[\frac{\sqrt{6}}{3}h,\frac{\sqrt{6}}{3}w\right]$. We expressed the multiple-layered similarity measure as follows

(10) $$S_{\mathrm{ml}}=\frac{1}{3}\times \sum_{1}^{3}S_{i}$$

where the similarity $S_{i=1,2,3}$ of each layer is calculated by using our proposed absent color indexing. ABC-ML is an image matching approach based on color histograms. In an image, pixels are counted as samples to generate the histogram. The number of statistical samples plays a crucial role in the accuracy of statistical analysis. Fewer statistical pixels can easily lead to the generated histogram cannot effectively represent the distribution of colors in the image. More layers can increase computational costs. The demerit of the histogram-based algorithm is that is difficult to obtain the position information of the color by statistics pixels. Therefore, the phenomenon of offset is easy to occur in the matching process, which leads to a decrease in the matching accuracy. By layering the template image, the color feature statistics of the relationship between each layer can indirectly add position information to solve the lack of the color histogram approach. For the above reason, images are divided into three layers. The pseudo-code of ABC-ML approach is shown in Algorithm 1.
**Algorithm** **1** Proposed ABC-ML approach.**Input:** Reference image $I_{r}$ and compared image $I_{s}$.           Extract each layer $I_{\kappa }^{r}$ and $I_{\kappa }^{s}$ of image $I_{r}$ and $I_{s}$, where κ=1,2,3.           Train the given dataset to determine the range of TCS.           Initial parameters: $\alpha =0.2,w_{D}=0.6,w_{N}=0.4,\beta_{1}=8,\beta_{2}=8,\kappa =1$.**Output:** Target location $L_{T}$ in the searched image.**repeat**   **repeat**     1: Generate two-dimensional color histograms $P_{\kappa }$ and $Q_{\kappa }$ by images $I_{\kappa }^{r}$ and $I_{\kappa }^{s}$ in TCS.     2: Divide color histograms into apparent color histograms $P_{\kappa }^{D}$, $Q_{\kappa }^{D}$ and absent color histograms $P_{\kappa }^{{}^{\prime }}$, $Q_{\kappa }^{{}^{\prime }}$.     3: Invert absent color histograms $P_{\kappa }^{{}^{\prime }}$ and $Q_{\kappa }^{{}^{\prime }}$ to $P_{\kappa }^{N}$ and $Q_{\kappa }^{N}$. κ=κ+1.   **until** κ>3;   4: Calculate each layer similarity $S_{\kappa }\left(x,y\right)$ by $P_{\kappa }^{D}$ and $Q_{\kappa }^{D}$, $P_{\kappa }^{N}$ and $Q_{\kappa }^{N}$ where κ=1,2,3.   5: The result of ABC-ML similarity at (x,y) position is $S_{\mathrm{ml}}\left(x,y\right)$.**until** All locations are scanned, then find position $L_{T}$ with $max(S_{\mathrm{ml}}\left(x,y\right))$;

## 5. Experiments

The structure of ABC-ML can be solved the problem of the shift of histogram-based approaches, which is without combining it with other features. In Section 5.1, the performance of our proposed ABC-ML is compared with color histogram-based approaches including color indexing (CI), cumulative color histogram method (CCH), and absent color indexing (ABC). Thereafter, template-based matching methods SSD, NCC, and BBS are utilized to illustrate the robustness and precision of ABC-ML in Section 5.2. To fair comparing, we set using experimental parameters $\beta_{1}=8,\beta_{2}=8,\alpha =0.2,w_{P}=0.6,w_{N}=0.4$ for all images or sequences in ABC-ML.

### 5.1. Performance Evaluation for Color Histogram-Based Methods

**Figure 10:**
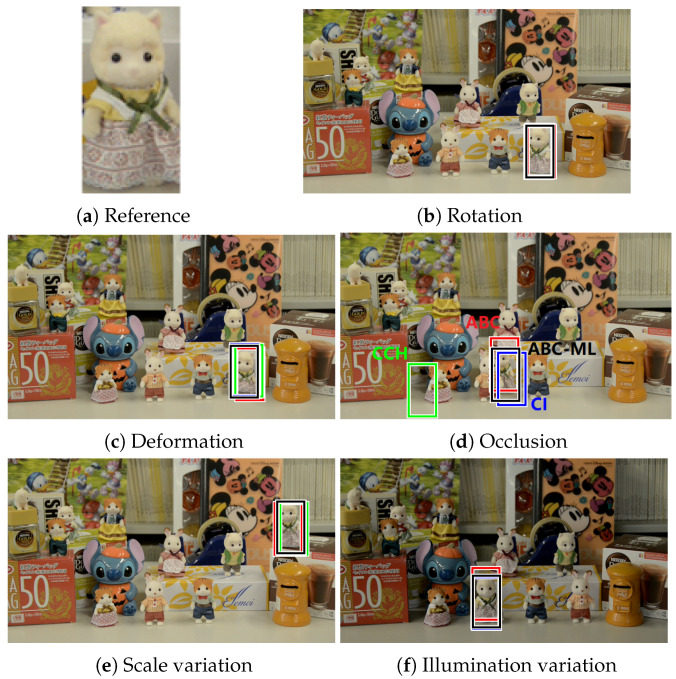
Comparison of color histogram-based matching methods. (**a**) Reference image. (**b**–**f**) show searching results by using different color histogram-based methods under different cases. Bounding blue, green, red, and black boxes are searching results by CI, CCH, ABC, and ABC-ML, respectively.

To provide a detailed explanation, we evaluate the experimental results of ABC-ML on real-world images. Five challenges are included, rotation, deformation, occlusion, scaling, and illumination variation, to observe the performance of our proposed approach with other color histogram-based approaches. The size of reference image in Figure 10a is 98×50 pixels. We set the same scene where the size is 360×640 pixels in the experiments. Euclidean distance is calculated to evaluate the performance of matching results. Among, the best-matched position from CI, CCH, ABC, and ABC-ML is compared with ground truth, respectively, to show the error in the matching process. In Figure 10, we can observe four color histogram approaches have a property of robustness. However, matching precision is unstable, which is easily appeared in the phenomenon of shift. Therefore, we improved our previous ABC approach for handling this problem, ABC-ML.

**Figure 11:**
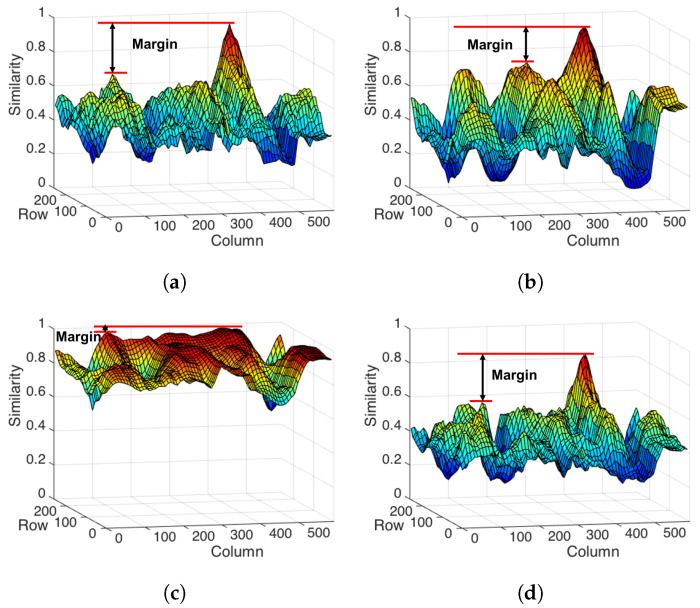
Analysis of profile plots which are used CI, CCH, ABC, and ABC-ML methods in Figure 10b. (**a**) The profile of similarity in ABC. (**b**) The profile of similarity in CI. (**c**) The profile of similarity in CCH. (**d**) The profile of similarity in ABC-ML.

**Figure 12:**
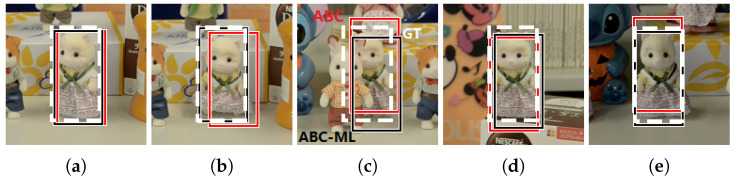
Comparison of matching precision between ABC and ABC-ML. ABC and ABC-ML results show in red and black bounding boxes. Ground truth is shown by white bounding box. (**a**) Rotation, (**b**) Deformation, (**c**) Occlusion, (**d**) Scale variation, (**e**) Illumination.

Under the case of rotation, four approaches can find the correct position of target image in the scene. After observing their profiles, the gap between the highest peak or the best-matched position and the second peak or matched position in our proposed ABC and ABC-ML approaches were larger than another two approaches as shown in Figure 11. Margin as a symbol represents a good distinguishability in image processing, especially in similar object matching. The matching results of ABC and ABC-ML are shown in Figure 12. White bounding boxes are ground truth. Observing the results, the matching location of ABC is roughly correct, but it has a shift. The matching results of ABC-ML are more precise. One reason is the position of the center point of each layer in ABC-ML is not changed; the other reason is we improved matching precision by the positional relationship and mutual control of each layer.

A comparison of the matching error of location is shown in Table 2. It is based on different color histogram approaches to challenges. Then, the location error of matching was calculated using the Euclidean distance, which is matched position to compare with the ground truth. The cases of rotation and occlusion, CI and ABC, can search for the best position in the experiments. In the other three cases, our proposed ABC-ML as it obtained the lowest error shows the best matching results.

### 5.2. ABC-ML Based Matching Analysis

Eight color histogram-based and template-based approaches are selected for comparison, which include SSD, NCC, CCH, CI, BBS, QATM, Siam R-CNN, and ABC. The comparison of matching results is described by location error. We calculate the Euclidean distance between the searched center- and ground truth center-point. We use the open data, Skiing data [27], to do experiments, and evaluate the performance of nine approaches. The skiing data are from OTB-2015. Images and ground truth are provided in this data. Among, ground truth is presented in the form of the location and size of the target. It includes challenges of illumination variation, scale variation, deformation, and rotation. In this sequence, a skier wearing a red ski coat and yellow ski pants slipped into the air and completed complicated movements such as flips. The scenes are dynamically changing and similar, mainly composed of the colors of trees, snow, and sky. For the skier, there have been scale variations from the beginning to the end of the sequence. There are a total of 81 frames in the data. The size of each frame is 360×640 pixels. In this experiment, reference image is 32×35 pixels. It contains a large amount of red and yellow, and a small amount of black. These color features can more intuitively reflect the different roles played by apparent and absent colors in the histogram. Furthermore, different challenges enable a more comprehensive evaluation of these contrasting algorithms. Therefore, skiing data are selected for evaluating the performance.

**Figure 13:**
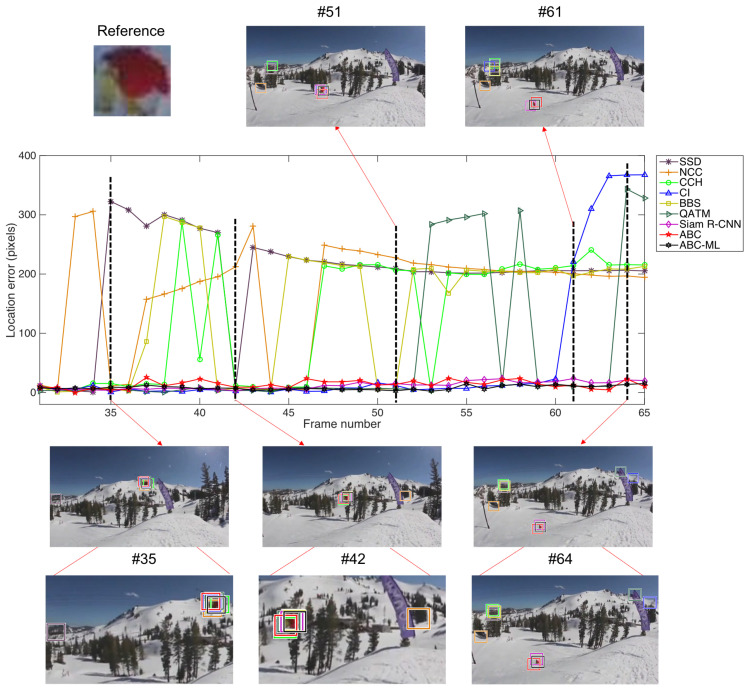
The matching performance of ABC-ML in Skiing data.

Figure 13 shows the tracking performance of ABC-ML in open data. The horizontal axis represents the video frame sequence. The vertical axis represents the location error by calculating the Euclidean distance between their best-matched positions and the ground truths. We use some representative frames as examples to show the difference in these nine approaches. In Frame #35, eight approaches can get good matching results, except SSD. Frame #42 shows NCC matched result is around the tree. Because template-based matching utilized the pixel value to calculate the matching result. In the scene, the similarities between correct and incorrect positions are almost identical. The challenges of deformation and scale variation are shown in Frames #61 and #64, where Siam R-CNN, ABC, and ABC-ML can match the target. The approaches of ABC and ABC-ML can catch absent or minor colors, which are red and yellow. The Siam R-CNN can find the target at the correct position as well. The difference between ABC-ML and Siam R-CNN is that ABC-ML searches pixel by pixel on each frame without relying on the information of the previous frame, while Siam R-CNN further combines the tracking track information of previous frames to predict the target position of the current frame. Because the skier is far away from the camera, background features occupy a large proportion. For another six approaches, it is hard to handle large deformation and scale variation. When the proportion of target colors is too small, for color histogram-based algorithm CI, background information in the scene is more matched than the correct position of the target. Therefore, CI cannot match the correct position of the target from Frame #61. Furthermore, the precision of ABC-ML is better than our prior proposed ABC approach.

To analyze the performance of our approach, we select four measurements as evaluation indicators, which include *Accuracy*, *Precision*, *Recall*, and *F-measure*. The formulas are defined as

(11) $$Accuracy=\frac{1}{N}\sum_{i=1}^{N}\frac{TP(i)+TN(i)}{TP(i)+TN(i)+FP(i)+FN(i)},$$

(12) $$Precision=\frac{1}{N}\sum_{i=1}^{N}\frac{TP(i)}{TP(i)+FP(i)},$$

(13) $$Recall=\frac{1}{N}\sum_{i=1}^{N}\frac{TP(i)}{TP(i)+FN(i)},$$

(14) $$F-measure=\frac{2\times Precision\times Recall}{Precision+Recall}.$$

where TP(i) and FP(i) are numbers of true and false positive pixels in frame *i*. TN(i) and FN(i) are numbers of true and false negative pixels in frame *i*. *N* is the total number of frames of the experiment. Table 3 lists the measure results of the performance of these nine approaches.

The ABC-ML approach demonstrated the best overall performance among all the approaches.

The computation cost of ABC-ML is calculated by using Visual Studio with OpenCV 4.4 library. For the hardware, a Windows 10 PC with a 2.2 GHz Intel Core i7-8750H CPU is used without parallel processing or GPU acceleration. A reference image with the size of 50×50 pixels was searched or matched pixel-wise in a scene. The size of the scene is 100×100 pixels. After searching, we obtained the entire computation cost which is 0.1865 s by the OpenCV timing function and 0.1866 s by the QueryPerformanceCounter function.

## 6. Conclusions

We propose a simple multiple-layered structure to improve the ABC method. ABC-ML learnt the color location information for robust and precise matching or tracking. The isotonic principle was used to control and define the structure of the three layers. Matching was performed using our proposed ABC approach. The original color histogram was decomposed into apparent and absent color histograms using threshold $h_{T}$. Absent colors, which are minor or nonexistent colors, are described to enhance distinguishability, especially for similar objects. The concept of the multiple-layered model provides strong support for overcoming the disadvantages of offset problems in color histogram-based approaches. The experimental results tested on real-world images and open data showed a high discrimination ability and robustness even under difficult conditions. The multiple-layered model can be extended to other application domains to roughly provide the position information and reduce the algorithm complexity. 

## Figures and Tables

**Table 1:** Value of $p^{N}$ in conditions of $p^{\prime }=0$.

$p^{N}$	$p^{\prime }=0,p>h_{T}$	$p^{\prime }=0,p=0$
q>0	0	$h_{T}$
q=0	0	0

**Table 2:** Location error in color histogram-based methods.

	CI	CCH	ABC	ABC-ML
Rotation	3.60	5.09	7.21	4.24
Deformation	3.60	11.04	14.31	2.23
Occlusion	29.06	158.1	12.04	14.86
Scale variation	11.40	16.27	11.04	9.21
Illumination variation	7	4.12	11.04	4

**Table 3:** Comparison of performance in different approaches.

	*Accuracy*	*Precision*	*Recall*	*F-Measure*
SSD	0.9927	0.1086	0.1154	0.1119
NCC	0.9930	0.1325	0.1468	0.1393
CCH	0.9940	0.2324	0.2694	0.2496
CI	0.9968	0.5072	0.7309	0.5988
BBS	0.9941	0.2717	0.2902	0.2806
QATM	0.9968	0.5007	0.7406	0.5975
Siam R-CNN	0.9969	0.5190	0.8399	0.6416
ABC	0.9955	0.3794	0.6285	0.4732
ABC-ML	0.9969	0.5138	0.8592	0.6431

## Data Availability

The data presented in this study are available upon reasonable request from the corresponding author.
